# Primary Central Nervous System Lymphoma With Systemic Recurrence

**DOI:** 10.7759/cureus.18406

**Published:** 2021-09-30

**Authors:** Sasirekha Pandravada, Phillip Knouse, Jacob Bitran

**Affiliations:** 1 Internal Medicine, Advocate Lutheran General Hospital, Park Ridge, USA; 2 Hematology and Medical Oncology, Advocate Lutheran General Hospital, Park Ridge, USA; 3 Hematology and Oncology, Advocate Lutheran General Hospital, Park Ridge, USA

**Keywords:** primary cns lymphoma, pcnsl, primary central nervous system lymphoma (pcnsl), oncology, central nervous system tumor

## Abstract

Primary central nervous system lymphoma (PCNSL) is an extranodal non-Hodgkin’s lymphoma confined to the brain, leptomeninges, spinal cord, or eyes without systemic involvement. Nearly half of patients with PCNSL who achieve complete remission, relapse within five years. The majority of patients who relapse have a local recurrence. Systemic relapse, however, is much rarer. Here, we report a rare case of a 70-year-old male diagnosed with PCNSL who relapsed systemically nearly 1.5 years after achieving complete remission. His treatment consisted of chemoimmunotherapy and targeted therapy followed by an autologous transplant. Currently, there is no standard of care for systemic relapse of PCNSL. This multiagent treatment modality may be one such option for salvage therapy.

## Introduction

Primary central nervous system lymphomas (PCNSLs) are extranodal non-Hodgkin’s lymphomas confined to the brain, spinal cord, eyes, or leptomeninges [1]. They are rare and account for 2-4% of central nervous system tumors among immunocompetent patients and less than 1% of non-Hodgkin lymphomas [1,2]. The vast majority of PCNSLs are diffuse large B-cell lymphomas, however, rarely they could be of other histologic types [1,2]. The median age of patients diagnosed with PCNSLs is 66 years, with more than 20% of patients older than 80 years at diagnosis [1,2]. The pathogenesis of the disease is incompletely understood but has been postulated to be due to either malignant transformation within the central nervous system or a systemic lymphoma that migrates to and preferentially grows within the central nervous system [3]. 

Though improved with the introduction of high-dose methotrexate-containing regimens, survival among patients with PCNSL lags behind that of systemic lymphomas [1,2]. The five-year survival rate is low in part because 35-60% of patients with PCNSL who achieve a complete response will relapse within five years [4,5]. Among those that relapse, the vast majority have CNS involvement while systemic relapse is much rarer [4]. Here, we report a case of a patient with PCNSL who developed the systemic disease at second relapse.

## Case presentation

**Figure 1:**
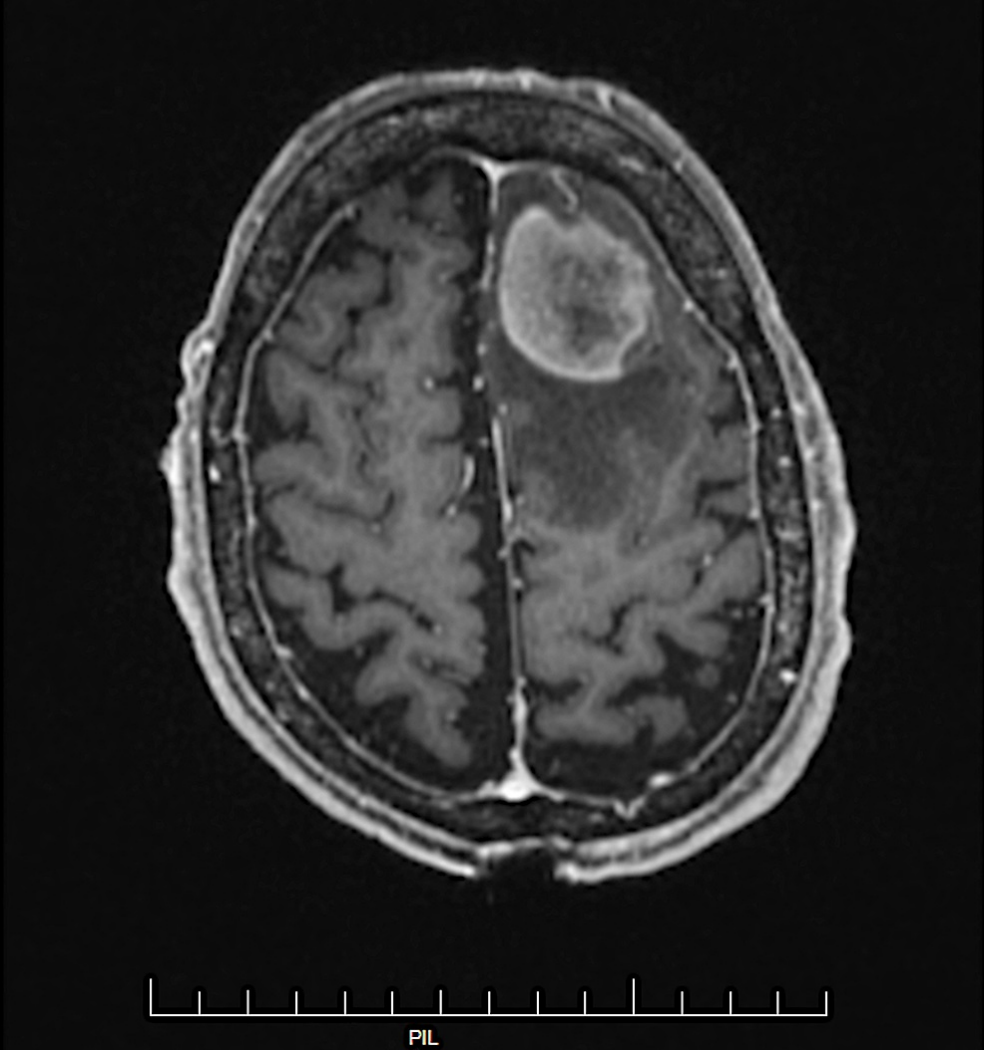
MRI brain displaying heterogeneous enhancement of a well-marginated 3.8 cm mass in the left frontal lobe anterosuperiorly with surrounding vasogenic edema.

**Figure 2:**
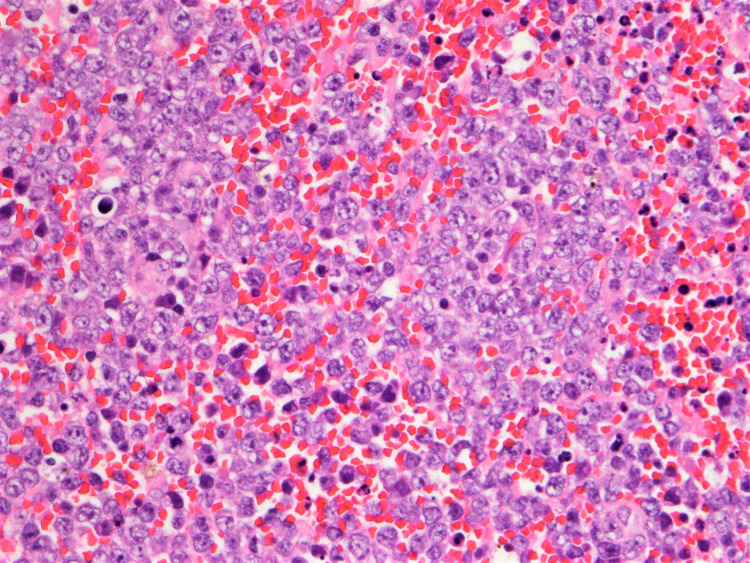
The brain biopsy demonstrates sheets of pleomorphic large lymphoid cells with H&E stain (400x) consistent with high-grade B-cell lymphoma.

A 70-year-old male presented in July 2017 following a syncopal episode. MRI brain confirmed a 3.8 cm mass in the left frontal lobe for which he underwent a craniotomy and gross total resection (Figure 1). Pathology was consistent with a high-grade B-cell lymphoma (Figure 2). Immunostaining was positive for cluster of differentiation (CD)-20, B-cell lymphoma antigen 6 (BCL6), glial fibrillary acidic protein (GFAP), and multiple myeloma oncogene-1 (MUM-1) and negative for CD3, CD10, and CD30. The Ki-65 index was 95%. There was no evidence of systemic lymphoma by PET/CT or bone marrow biopsy, consistent with PCNSL.

In August 2017, the patient initiated induction therapy with high-dose methotrexate and rituximab. In October 2017, he was confirmed to have progressive disease after three cycles of methotrexate and rituximab for which he received one cycle of salvage nivolumab with no response. He underwent repeat resection and 15 fractions of external beam radiation therapy with a partial response, though in January 2018 repeat MRI brain confirmed disease progression. In February 2018, he initiated ibrutinib 420 mg daily for 12 months with which he achieved complete remission. Ibrutinib was ultimately discontinued due to debilitating musculoskeletal pain, a rarer side effect of therapy, though serial surveillance MRIs confirmed a sustained response.

**Figure 3:**
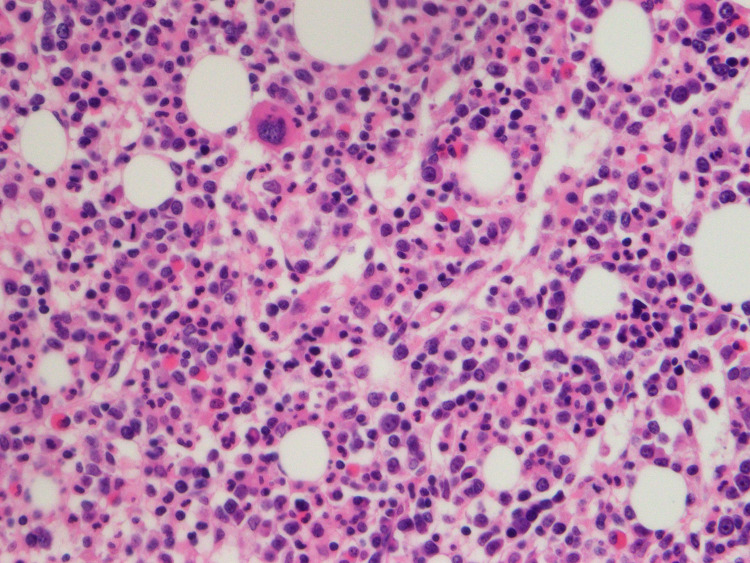
Bone marrow biopsy, H&E stain, CD20 immunostain 400x. The bone marrow biopsy demonstrates many large pleomorphic lymphoid cells highlighted by CD20 immunostain with H&E stain consistent with hypercellular bone marrow extensively involved by large B-cell lymphoma. CD: cluster of differentiation

**Figure 4:**
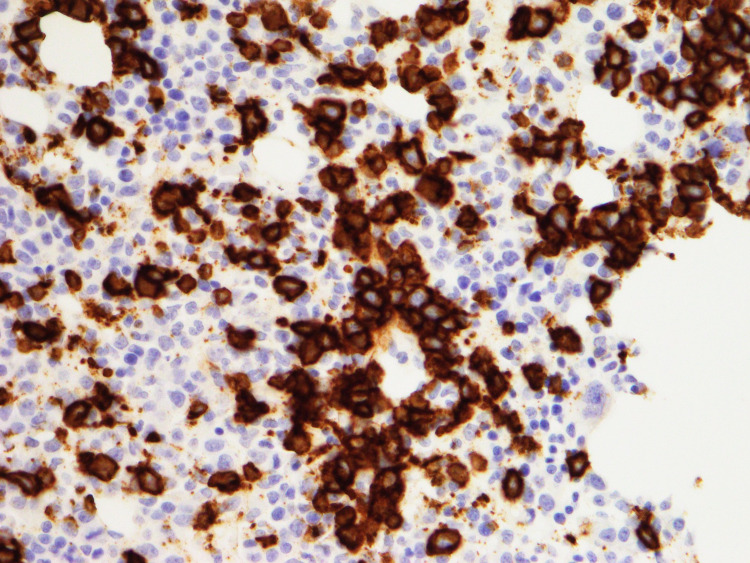
Bone marrow biopsy, CD20 stain 400x This bone marrow biopsy demonstrates numerous large pleomorphic CD20 positive lymphoid cells consistent with systemic relapse primary CNS lymphoma. CD: cluster of differentiation; CNS: central nervous system

In July 2020, he presented again with fever, cough, and pancytopenia. CT scan at that time showed moderate splenomegaly measuring 16.5 cm (compared to 13.2 cm on initial presentation) but was otherwise unremarkable. Bone marrow biopsy confirmed a high-grade B-cell lymphoma, consistent with systemic relapse of PCNSL (Figures 3, 4). Salvage therapy consisted of intrathecal methotrexate and six cycles of rituximab, cyclophosphamide, doxorubicin, vincristine, and prednisone (R-CHOP). Post-treatment PET/CT and MRI confirmed complete response. He underwent consolidative autologous stem cell transplant and is now more than 100 days from transplant without evidence of relapse.

## Discussion

The prognosis of patients with relapsed PCNSL is poor and a standard of care for these patients has not yet been defined. This is certainly true for those with extracranial systemic relapse as systemic relapse is especially uncommon [6].

Jahnke et al., in 2006, reported seven systemic relapses among a cohort of 51 patients with PCNSL, two of whom had bone marrow involvement at relapse [4]. Similarly, Provencher et al., in 2011, described a case series of 209 patients with PCNSL, 10 (4.8%) of whom had systemic relapse. Interestingly, Provencher et al.'s case series suggest that those with systemic relapse may fare better than those who relapse within the CNS. In their study, the median overall survival for those with systemic relapse was 15.5 months compared to 4.6 months for those who relapsed within the CNS [7]. These data should be interpreted with caution, however, because of their small cohort size.

Our patient presented with moderate splenomegaly of unclear cause at original diagnosis. PET/CT scan and bone marrow biopsy failed to reveal evidence of systemic disease and he was diagnosed with PCNSL. Occult systemic disease beyond the level of detection of PET/CT is theoretically possible, but it is unlikely as the systemic recurrence occurred nearly 1.5 years from his original diagnosis.

Interestingly, our patient achieved a complete response to ibrutinib after the first relapse, and this response was sustained for more than one year. Data from the phase II iLOC study published in 2019 demonstrated an impressive 69% disease control rate and 19% complete response rate with ibrutinib monotherapy among patients with relapsed and refractory PCNSL [8]. Ongoing trials involving ibrutinib with combination therapy are also showing promising results. In a phase Ib trial involving the combination of ibrutinib with high dose methotrexate and rituximab, 11 of 15 patients proceeded to maintenance with ibrutinib after completing four cycles of combination therapy [9]. Newer trials involving immunotherapy are underway, including ongoing phase Ib/II trials evaluating ibrutinib in combination with PD-1 inhibitors, such as nivolumab and pembrolizumab [10,11]. Our experience provides additional evidence that Bruton tyrosine kinase inhibitors can be one such treatment option for patients with refractory or relapsed PCNSL.

## Conclusions

Systemic relapse of primary CNS lymphoma is rare and currently has a poor prognosis. The addition of multiagent chemotherapy to ibrutinib has shown promise in early trials. Further ongoing trials with ibrutinib in combination with immunotherapy also highlight the potential role of Bruton tyrosine kinase inhibitors. As with our patient, ibrutinib along with multiagent chemoimmunotherapy followed by autologous transplant may be an effective salvage strategy in those with refractory or relapsed PCNSL.

## References

[1] Liu Y, Yao Q, Zhang F (2021). Diagnosis, prognosis and treatment of primary central nervous system lymphoma in the elderly population (review). Int J Oncol.

[2] Song KW, Issa S, Batchelor T (2021). Primary central nervous system lymphoma: epidemiology and clinical presentation. Ann Lymphoma.

[3] Smith JR, Braziel RM, Paoletti S, Lipp M, Uguccioni M, Rosenbaum JT (2003). Expression of B-cell-attracting chemokine 1 (CXCL13) by malignant lymphocytes and vascular endothelium in primary central nervous system lymphoma. Blood.

[4] Jahnke K, Thiel E, Martus P, Herrlinger U, Weller M, Fischer L, Korfel A (2006). Relapse of primary central nervous system lymphoma: clinical features, outcome and prognostic factors. J Neurooncol.

[5] Nayak L, Hedvat C, Rosenblum MK, Abrey LE, DeAngelis LM (2011). Late relapse in primary central nervous system lymphoma: clonal persistence. Neuro Oncol.

[6] Ahmed Z, Ramanathan RK, Ram S, Newell J, Halepota M (2014). Unusual relapse of primary central nervous system lymphoma at site of lumbar puncture. Case Rep Hematol.

[7] Provencher S, Ferlay C, Alaoui-Slimani K (2011). Clinical characteristics and outcome of isolated extracerebral relapses of primary central nervous system lymphoma: a case series. Hematol Oncol.

[8] Soussain C, Choquet S, Blonski M (2019). Ibrutinib monotherapy for relapse or refractory primary CNS lymphoma and primary vitreoretinal lymphoma: final analysis of the phase II 'proof-of-concept' iLOC study by the lymphoma study association (LYSA) and the French oculo-cerebral lymphoma (LOC) network. Eur J Cancer.

[9] Grommes C, Tang SS, Wolfe J (2019). Phase 1b trial of an ibrutinib-based combination therapy in recurrent/refractory CNS lymphoma. Blood.

[10] Nayak L. (2020, Aug 1 (2021). Pembrolizumab, ibrutinib and rituximab in PCNS. https://clinicaltrials.gov/ct2/show/NCT04421560.

[11] (2021). Nivolumab and ibrutinib in treating patients with relapsed or refractory central nervous system lymphoma. https://clinicaltrials.gov/ct2/show/NCT03770416?term=ibrutinib&cond=Primary+CNS+Lymphoma&draw=2&rank=7.

